# Parallel sequencing of 170 STR and 132 SNP markers using the FGID forensic four-in-one DNA typing kit on the DNBSEQ-G99RS platform

**DOI:** 10.1093/fsr/owae050

**Published:** 2024-08-21

**Authors:** Xiaoyuan Zhen, Zhenmin Zhao, Ruocheng Xia, Xiling Liu, Hui Li, Yuzhen Gao, Baifang He, Chengtao Li, Ruiyang Tao

**Affiliations:** Shanghai Key Laboratory of Forensic Medicine, Shanghai Forensic Service Platform, Academy of Forensic Sciences, Key Laboratory of Forensic Science, Ministry of Justice, Shanghai, China; College of Forensic Science, School of Medicine, Xi’an Jiaotong University, Xi’an, China; Shanghai Key Laboratory of Forensic Medicine, Shanghai Forensic Service Platform, Academy of Forensic Sciences, Key Laboratory of Forensic Science, Ministry of Justice, Shanghai, China; Shanghai Key Laboratory of Forensic Medicine, Shanghai Forensic Service Platform, Academy of Forensic Sciences, Key Laboratory of Forensic Science, Ministry of Justice, Shanghai, China; Shanghai Key Laboratory of Forensic Medicine, Shanghai Forensic Service Platform, Academy of Forensic Sciences, Key Laboratory of Forensic Science, Ministry of Justice, Shanghai, China; Shanghai Key Laboratory of Forensic Medicine, Shanghai Forensic Service Platform, Academy of Forensic Sciences, Key Laboratory of Forensic Science, Ministry of Justice, Shanghai, China; Departments of Forensic Medicine, Medical College of Soochow University, Suzhou, China; BGI Forensic Technology Co., Ltd, Shenzhen, China; Shanghai Key Laboratory of Forensic Medicine, Shanghai Forensic Service Platform, Academy of Forensic Sciences, Key Laboratory of Forensic Science, Ministry of Justice, Shanghai, China; Shanghai Medical College, Fudan University, Shanghai, China; Shanghai Key Laboratory of Forensic Medicine, Shanghai Forensic Service Platform, Academy of Forensic Sciences, Key Laboratory of Forensic Science, Ministry of Justice, Shanghai, China

**Keywords:** forensic sciences, forensic genetics, massive parallel sequencing, DNBSEQ-G99RS, STRs, SNPs

## Abstract

Massive parallel sequencing (MPS) has rapidly emerged as a promising technique for forensic DNA typing due to its capacity to simultaneously detect numerous genetic markers and samples in a single reaction, allowing the direct acquisition of sequence information. In this current investigation, the FGID forensic four-in-one DNA typing kit was employed on the DNBSEQ-G99RS high-throughput sequencing platform to simultaneously analyse two types of forensic genetic markers—short tandem repeat (STR) and single nucleotide polymorphism (SNP). A total of 306 DNA markers, comprising Amelogenin, 66 autosomal STR (A-STR) loci, 29 X chromosomal STR (X-STR) loci, 75 Y chromosomal STR (Y-STR) loci, and 135 SNP (132 A-SNP and 3 Y-SNP) loci, were genotyped for 100 unrelated individual samples (50 males and 50 females). As a result, sequence-based STR typing identified 940 alleles on A-STRs, 378 alleles on X-STRs, and 519 alleles on Y-STRs. In comparison with length-based alleles, the number of unique alleles based on sequence increased by 58.18%. Additionally, 97 new sequence variations were observed at 29 STR loci, and MPS sequence information was obtained for the first time at 42 STR loci. Furthermore, when utilizing sequence-based data, forensic parameters exhibited a notable increase in combined power of discrimination (CPD) and combined power of exclusion for A-STR, a slight increase in CPD and combined mean exclusion chance for X-STR, and a marginal increase in discrimination capacity for Y-STR. Moreover, information data for 132 A-SNPs were acquired. As anticipated, our findings highlight the advantages of MPS in forensic genetic applications while contributing novel genetic data for Asian populations in forensic practice.

## Introduction

Short tandem repeats (STRs) as a potent tool have the capability to generate diverse combinations of genotypes, contributing to genetic diversity across various populations or species. They are commonly employed in genetic profiling and forensic research [1]. Traditionally, gene typing utilizing STR markers heavily relied on polymerase chain reaction (PCR) and capillary electrophoresis (CE) [2]. Additionally, single nucleotide polymorphisms (SNPs), mainly situated in non-coding regions of autosomal chromosomes, serve as valuable supplementary markers due to their advantages such as a low mutation rate, and small amplicon size [3]. However, the limitations of the CE method become apparent as the diversity and complexity of case samples increase. Furthermore, the restricted number of genetic polymorphism markers utilized in PCR-CE methods [4, 5] leads to insufficient effectiveness in certain specialized kinship analyses. Simultaneously genotyping a substantial number of STRs and SNPs in a single assay becomes impractical [6].

Next-generation sequencing (NGS), also recognized as massive parallel sequencing (MPS), enables the simultaneous sequencing of millions of DNA fragments, producing a wealth of intricate nucleotide sequences and valuable sequencing data pertaining to potential variations in flanking regions (FRs). This technology facilitates the incorporation of a larger number of genetic markers into multiplex systems, resulting in a notable enhancement in the polymorphism information [7]. Since its inception in 2005, MPS has made significant advancements in the field of forensic science, showcasing numerous potential advantages [7–9]. In 2015, Illumina introduced the MiSeq FGx Forensic Genomics System (referred to as MiSeq FGx) along with the ForenSeq DNA Signature Prep Kit, both extensively validated and assessed in diverse forensic applications worldwide [10–13]. By leveraging MPS technology and appropriate forensic genetic markers, it also becomes feasible to extract a greater volume of relevant information from samples with limited quantity and quality.

Recently, a set of panels based on MPS has been developed. These panels cover autosomal STRs (A-STRs), sex chromosomal STRs (X-STRs, Y-STRs), identity, phenotypic, and ancestry-informative SNPs (iiSNPs, piSNPs, aiSNPs), as well as mitochondrial DNA (mtDNA) markers. They have undergone a series of validations [12, 14–18]. These innovative forensic panels hold greater potential for conducting individual identification, intricate kinship analysis, and lineage identification in forensic practice. In the context of applications across major global sequencing platforms, FGI Tech (MGI, Shenzhen, China) provides a promising alternative among MPS platforms, by employing unique DNA nanoball (DNB) technology which combines single-stranded circular (ssCir) library construction, generation and loading of DNBs onto optimized flow cell, and combinatorial probe-anchor synthesis (cPAS) sequencing. Previous studies have shown that the MGI platform exhibits a comparable accurate rate and sequencing quality compared with the Illumina platform, as verified by a range of studies [18–21].

The DNBSEQ-G99RS platform (MGI Tech) represents the latest addition to FGI Tech’s lineup of NGS machines, following the BGISEQ-500 and MGISEQ-2000. Recently, the FGID forensic four-in-one DNA typing kit (MGI Tech) has been specifically crafted for forensic applications based on the DNBSEQ-G99RS platform. This innovative kit encompasses Amelogenin, 170 STRs and 135 SNPs, covering autosomal, X-chromosomal, and Y-chromosomal makers (including 66 A-STRs, 29 X-STRs, 75 Y-STRs, 132 A-SNPs, and 3 Y-SNPs). Additionally, it includes seven amplicons that span the entire hypervariable region (HVR 1 to HVR 7) of the mtDNA. In this study, leveraging the DNBSEQ-G99RS platform, forensic genetic analysis was conducted on a Chinese Han population comprising 100 unrelated individuals.

## Materials and methods

### Sample preparation

Following the protocol approved by the Ethics Committee of the Academy of Forensic Science, Ministry of Justice of the People’s Republic of China, peripheral blood samples were obtained from 100 unrelated individuals of Han Chinese ethnicity, with their written informed consent. Genomic DNA from human peripheral blood was extracted using the QIAamp DNA Blood Mini Kit (Qiagen, Hilden, Germany). Subsequently, quantification was performed in accordance with the manufacturer’s instructions, utilizing the Qubit® dsDNA HS Assay Kit and a Qubit® 2.0 Fluorometer (Thermo Fisher Scientific, Waltham, MA, USA).

### Library construction

In general, library preparation was carried out using an FGID forensic four-in-one DNA typing kit (MGI, Shenzhen, China) following the manufacturer’s recommendations.

The first round of multiplex PCR reactions was conducted to obtain DNA products from the target region: approximately 2 ng of genomic DNA under the following thermal cycling conditions: activation at 95°C for 5 min, followed by 20 cycles of denaturation at 95°C for 15 s, annealing at 58°C for 3 min, and extension at 72°C for 30 s. The final extension step was performed at 72°C for 10 min, followed by storage at 4°C.

The objective of the second round of PCR reactions was to assign a distinctive Barcode ID to each sample. Following both rounds of PCR reactions, the PCR products from each sample underwent purification. The purification process could be executed using the MGISP-960 High-Throughput Automated Sample Preparation System (MGI). Subsequently, the barcode DNA libraries were combined in an equimolar manner to achieve a total DNA amount of 350 ng. The DNA libraries underwent mixing, denaturation, looping, and additional enzyme-purification to transform them into single-stranded circular DNA libraries. The concentration of the single-stranded DNA (ssDNA) fragments was assessed using the Qubit® ssDNA HS Assay Kit on a Qubit® 2.0 Fluorometer (Thermo Fisher Scientific), following the manufacturer’s protocols.

### Make DNB and sequencing

DNBs were produced from these single-stranded circular DNA libraries *via* the process of rolling circle amplification (RCA). Subsequently, these DNBs were loaded onto flow cells through the utilization of cPAS before undergoing sequencing.

To prepare the DNB library, we started with 5 ng of ssDNA from the library. Then, we added TE buffer to make the final volume 10 μL. Next, we add 10 μL of DNB preparation buffer. The PCR reaction conditions were as follows: incubation at 95°C for 1 min, and at 65°C for 1 min, incubate at 40°C for 1 min. After reaching 40°C, we added 20 μL of “make DNB enzyme Mix” and 2 μL of “make DNB enzyme Mix II”. Then, we incubated for an additional 20 min at 30°C. Afterwards, when the temperature dropped to 4°C, we added 10 μL of DNB stop buffer, mixed well, and quantified the sample. Next, we took 10 μL of the DNB, added 7 μL of DNB load buffer II, and 1 μL of “make DNB enzyme Mix II”. Subsequently, a sequencing kit sourced from MGI was utilized to prepare and load nanospheres onto the chip. The MPS was conducted on the DNBSEQ-G99RS platform employing a single-end 400 strategy, in accordance with the manufacturer’s guidelines. Three runs were performed, with each sequencing run analysing either 48 or 32 libraries, and including one positive and one negative control.

### CE-typing

To confirm the genotype of D18S853 and D1S1627 loci in multiple samples, CE-STR genotyping was performed using AGCU 21 + 1 STR kit (AGCU ScienTech, Wuxi, China). Adhering to the manufacturer’s guidelines, DNA quantification was executed, and PCR amplification was carried out using the GeneAmp PCR System 9700 (Applied Biosystems, Foster City, CA, USA). The resulting amplification products are subsequently separated and detected using an Applied Biosystems 3500 DNA Analyzer (Thermo Fisher Scientific). Following this, the CE raw data were subjected to analysis using GeneMapper ID-X software v1.5 (Thermo Fisher Scientific).

### Data analysis and statistics

This study followed the validation principles outlined by the Scientific Working Group on DNA Analysis Methods (SWGDAM) (https://www.swgdam.org). The valid read counts for all loci were extracted from XLSX files produced by the detailed sample information report of the FGID forensic DNA analysis system. The depth of coverage (DoC) was established by summing up all the valid reads at a specific locus. FR variations of STRs were examined with respect to the GRCh38 genome. Alleles were designated following the nomenclature guidelines proposed by the International Society for Forensic Genetics (ISFG) [22].

PowerStats version 2.1 (Promega, Madison, WI, USA) was utilized for the computation of various forensic parameters, encompassing allele frequencies, power of discrimination (PD), polymorphism information content (PIC), power of exclusion (PE), match probability (MP), and typical paternity index. Arlequin suite v3.5 [23], a software specializing in population genetics analysis, was employed to assess deviations from Hardy–Weinberg equilibrium (HWE) and conduct pairwise linkage disequilibrium (LD) tests between autosomal loci situated on the same chromosome. In the case of X-STRs, the Forensic ChrX Research database (https://www.chrx-str.org) was employed to calculate PIC, the power of discrimination for males and females (PD_M_ and PD_F_, respectively), as well as mean exclusion chance (MEC) in deficiency cases and standard trios following Krüger and Püschel [24] (MEC_Krüger_ and MEC_Kishida_), and MEC in standard trios and duos (MEC_Desmarais_ and MEC_Desmarais Duo_, respectively). Regarding Y-STRs, haplotype diversity (HD) and genetic diversity (GD) were computed by direct counting using the following formulas:


\begin{equation*} \mathrm{HD}=\frac{N\times \left(1-{\sum}_{{i}=1}^{{k}}{\mathrm{p}}_{{i}}^2\right)}{N-1}, \end{equation*}

\begin{equation*} \mathrm{DC}=\frac{{k}}{\sum_{{i}=1}^{{k}}\left({\mathrm{p}}_{{i}}\times N\right)}, \end{equation*}

where p_*i*_ represents the frequency of the *i*th haplotype, *k* denotes the total number of haplotypes, and *N* indicates the overall sample size [25]. All forensic parameters were computed in accordance with the Specification of Paternity Testing issued by the Ministry of Justice the People’s Republic of China (SF/ZJD0105001-2016).

## Results

### Sequencing quality

For three runs, the total reads were up to 104.9 million per sequencing run. The average of Quality score 30 (Q30) for the three runs was 56.76%, while the first 100 cycle Q30 ratio reached >89.06%. Each run demonstrated metrics falling within acceptable ranges, covering both positive and negative control samples. Within the set of 100 unrelated individual samples, the average DoC for 170 STRs was 8 140×, with A-STR, X-STR, and Y-STR having average DoCs of 7 449×, 15 525×, and 6 011×, respectively. The range of average DoC for STRs spanned from 486× for DYS616 to 145 175× for DYS385a/b, with the highest-to-lowest ratio being 299. Among these, seven STRs (vWA, D1S1656, CSF1PO, DXS10103, DYS481, Y-GATA-H4, and DYS460) fell below 650×. Concerning SNPs, the average DoC ranged from 29× at rs1736442 to 1 478× at rs1109037.

### Alleles information

**Figure 1:**
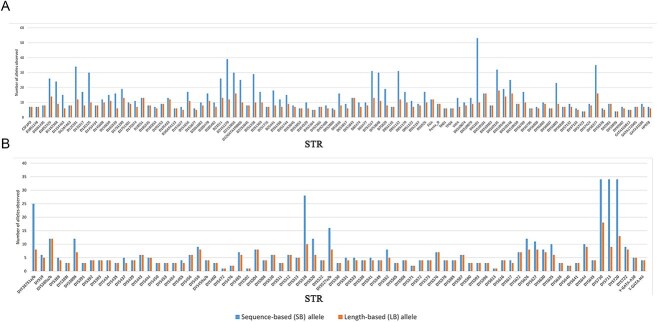
Sequence-based (SB) and length-based (LB) unique allele counts at each locus from 100 unrelated Chinese Han individuals. (A) A-STR and X-STR. (B) Y-STR.

Figure 1 presents the count of sequence-based (SB) and length-based (LB) unique alleles from 100 unrelated Han Chinese individuals. Null alleles at DYS644 in one sample was neglected, and we refrained from verification by CE due to DYS644 not being covered in any commercial kits. Across 170 STRs, MPS typing detected 1 837 alleles, whereas CE typing yielded 1 198 LB alleles (Supplementary Table S1). The overall increase in the number of unique alleles was 58.18% with SB alleles compared with LB alleles. DXS101 had the highest number of SB alleles (53), followed by D21S1270 (39), which also displayed high diversity (PIC: 0.967, 0.831). The sequences and frequencies of all STR alleles are detailed in Supplementary Tables S2–S4. The MPS analysis of these STRs significantly enhanced allele diversity, consistent with findings in previous studies [10–12, 26–41].

Variants were only detected within the repeat region (RR) at 44 loci, including 24 A-STRs (D1S1656, D1S1677, D2S1360, D3S1358, D3S1744, D3S4529, D4S2408, D5S2800, D6S477, D7S3048, D8S1132, D8S1179, D9S1122, D9S925, D11S4463, D12S391, D14S608, D15S659, D17S1290, D17S1301, D18S853, D21S11, D21S2055, and D22GATA198B), five X-STRs (DXS10074, DXS10135, DXS7133, DXS8377, and GATA31E08), and 15 Y-STRs (DYS19, DYS389II, DYS437, DYS458, DYS518, DYS520, DYS527a/b, DYS533, DYS541, DYS552, DYS630, DYS635, DYS644, DYS710, and DYF387S1a/b), and the number of unique alleles increased from 11% (D17S1301) to 213% (DYS387S1a/b). Variants were only detected within the FR at 11 A-STRs (D16S539, D17S974, D1S1627, D20S1082, D20S470, D21S1270, D3S3045, D5S818, D6S1017, D6S474, and D9S2157), six X-STRs (DXS6800, DXS6803, DXS7132, DXS7424, DXS8378, and DXS9902), and six Y-STRs (DYS388, DYS455, DYS531, DYS626, DYS627, and DYS439), and the number of unique alleles increased from 13% (D9S2157) to 225% (D21S1270). Either RR or FR variants were observed at 14 loci, including eight A-STRs (D10S2325, D11S2368, D13S317, D20S482, D2S1338, D7S1517, D7S820, and vWA), four X-STRs (DXS10079, DXS101, DXS10146, and DXS6809), and two Y-STRs (DYS713 and DYS720), and the number of unique alleles increased from 8% (Penta D) to 167% (D11S2368). We observed that variants in the RR constituted the predominant source of variations leading to the augmented number of alleles. These findings align closely with, albeit displaying slight distinctions from, those reported in prior studies [10, 26–30, 35, 36, 38, 40, 41].

### Novel alleles and sequence variations

The panel in this study encompasses a broader spectrum of chromosomal genetic loci, encompassing not only typical CODIS loci but also some non-CODIS loci. In the characterization of distinctive alleles identified in this investigation, various intriguing sequence variations were uncovered. In this study, some loci (D4S2364, D8S1115, D21S2055, DXS10148, DXS6789, DXS7133, DXS7424, DXS981, DXS9895, DXS9902, GATA165B12, GATA172D05, GATA31E08, DYS434, DYS435, DYS450, DYS453, DYS454, DYS455, DYS472, DYS476, DYS502, DYS511, DYS512, DYS513, DYS530, DYS538, DYS541, DYS565, DYS568, DYS571, DYS572, DYS573, DYS578, DYS585, DYS590, DYS613, DYS616, DYS638, DYS640, DYS713, and DYS722) were first researched using MPS (Supplementary Table S2), and the sequence details have not been documented in the published MPS system.

By contrasting the STR Sequencing Project [42] and previous studies [10, 11, 26–41], a total of 97 novel RR variant alleles were found at 29 STR (D11S2368, D11S4463, D13S325, D14S1434, D18S51, D18S853, D1S1677, D20S470, D20S482, D3S1744, D7S1517, D7S3048, D8S1132, D8S1179, D15S659, D17S1290, D21S1270, DXS10074, DXS10135, DYS387S1a/b, DYS458, DYS485, DYS518, DYS527a/b, DYS587, DYS626, DYS644, DYS645, and DYS710), summarized in Table 1. The common situation is that the variety of repeat numbers occurs when there are compound or complex repeats; to be specific, alleles [TGTC]1 [TATC]13 and [TGTC]1 [TATC]14 of D11S4463, alleles [CTTT]2 [GTTT]1 [CTTT]2 [GTTT]2 [CTTT]12 and [CTTT]2 [GTTT]1 [CTTT]2 [GTTT]2 [CTTT]13 of D7S1517, alleles [AAAG]16 [AG]14 [AAAG]9 and [AAAG]16 [AG]14 [AAAG]10 of DYS710. In addition, the mutation happening in the core sequence, such as allele [TCTA]8 TCA [TCTA]11 and [TCTA]7 TCA [TCTA]10 [TCTG]1 [TCTA]1 of D8S1132, allele [TATC]1 ctttctccataat [TATC]13 [TACC]8 and [TATC]1 ctttctccataat [TATC]12 [TACC]7 [CACC]2 of D7S3048.

**Table 1:** Novel repeat region (RR) variant alleles identified in this study (*N* = 100).

Locus	Allele	Motif	Count	Frequency	Locus	Allele	Motif	Count	Frequency
D11S2368	18	[TATC]3 [TGTC]5 [TATC]10	1	0.005	D8S1132	14.1	[TCTA]13 TCTG [TCTA]1	3	0.015
	19	CATC [TATC]2 [TGTC]2 [TATC]14	1	0.005		19	[TCTA]8 TCA [TCTA]11	1	0.005
	21	[TATC]3 [TGTC]3 [TATC]15	2	0.01		19	[TCTA]7 TCA [TCTA]10 [TCTG]1 [TCTA]1	1	0.005
D11S4463	12	[TGTC]1 [TGTC]1 [TATC]10	1	0.005		20	[TCTA]10 TCA [TCTA]8 TCTG [TCTA]1	1	0.005
	13	[TGTC]1 [TGTC]1 [TATC]11	2	0.01		20	[TCTA]9 TCA [TCTA]9 TCTG [TCTA]1	1	0.005
	14	[TGTC]1 [TGTC]1 [TATC]12	1	0.005		21.3	[TCTA]8 TCA [TCTA]3 TCA [TCTA]8	1	0.005
D13S325	17	[TCTA]7 TCA [TCTA]10	2	0.01			[TCTG]1 [TCTA]1		
	18	[TCTA]7 TCA [TCTA]11	2	0.01	D21S1270	10.3	[ATAG]3 ATG [ATAG]1 ATG [ATAG]1 ATG	1	0.005
	18	[TCTA]9 TCA [TCTA]9	4	0.02			[ATAG]5		
	19	[TCTA]10 TCA [TCTA]9	6	0.03	D7S1517	15	[CTTT]2 [GTTT]1 [CTTT]2 [GTTT]3 [CTTT]7	2	0.01
	19	[TCTA]11 TCA [TCTA]8	1	0.005		17	[CTTT]2 [GTTT]1 [CTTT]2 [GTTT]3 [CTTT]9	1	0.005
	19	[TCTA]8 TCA [TCTA]11	1	0.005		19	[CTTT]2 [GTTT]1 [CTTT]2 [GTTT]4 [CTTT]10	1	0.005
	20	[TCTA]8 TCTG [TCTA]1 TCA [TCTA]10	1	0.005		20	[CTTT]2 [GTTT]1 [CTTT]2 [GTTT]2 [CTTT]13	4	0.02
	22	[TCTA]10 TCA [TCCA]1 [TCTA]11	1	0.005		23	[CTTT]2 [GTTT]1 [CTTT]2 [GTTT]5 [CTTT]13	3	0.015
	23	[TCTA]4 [TATA]1 [TCTA]9 TCA	1	0.005		25	[CTTT]2 [GTTT]1 [CTTT]2 [GTTT]5 [CTTT]15	1	0.005
		[TCTA]9				26	[CTTT]2 [GTTT]1 [CTTT]2 [GTTT]1 [CTTT]2	1	0.005
	24	[TCTA]11 TCA [TCTA]13	1	0.005			[GTTT]1 [CTTC]1 [CTTT]1 [GTTT]1 [CTTT]2		
	24	[TCTA]12 TCA [TCTA]12	1	0.005			[GTTT]1 [CTTT]2 [GTTT]1 [CTTT]8		
	24	[TCTA]13 TCA [TCTA]11	2	0.01		27	[CTTT]2 [GTTT]1 [CTTT]2 [GTTT]1 [CTTT]2	4	0.02
	25	[TCTA]14 TCA [TCTA]11	1	0.005			[GTTT]1 [CTTC]1 [CTTT]1 [GTTT]1 [CTTT]2		
D8S1179	19	[TCTA]2 [TCTG]1 [TCTA]16	1	0.005			[GTTT]1 [CTTT]2 [GTTT]1 [CTTT]9		
D15S659	13	[TATC]7 [TACC]1 [TATC]5	1	0.005	D17S1290	13	[AGAT]4 GATG [ATAG]15	1	0.005
	14	[TATC]8 [TACC]1 [TATC]5	1	0.005		15.2	[AGAT]4 GATG [ATAGATAT]3 [ATAG]8 AT	1	0.005
	16	[TATC]16	1	0.005			[ATAG]3		
D1S1677	14	[TTCC]1 TTCT [TTCC]12	1	0.005	DYS710	30.2	[AAAG]16 [AG]11 [AAAG]9	1	0.02
D20S470	8	[AGGA]8	1	0.005		32	[AAAG]16 [AG]14 [AAAG]9	3	0.06
D20S482	12	[AGAT]8 AGTT [AGAT]3	1	0.005		33	[AAAG]16 [AG]14 [AAAG]10	1	0.02
D17S1290	13	[AGAT]4 GATG [ATAG]15	1	0.005		33.2	[AAAG]17 [AG]11 [AAAG]11	2	0.04
	15.2	[AGAT]4 GATG [ATAGATAT]3	1	0.005		34	[AAAG]15 [AG]12 [AAAG]13	4	0.08
		[ATAG]8 AT [ATAG]3				34	[AAAG]17 [AG]12 [AAAG]11	2	0.04
D18S51	17.1	[AGAA]5 A [AGAA]12	1	0.005		34.2	[AAAG]13 [AG]13 [AAAG]15	1	0.02
D18S853	11	[ATA]10 AAA	1	0.005		34.2	[AAAG]18 [AG]13 [AAAG]10	2	0.04
D14S1434	13.3	[CTGT]3 [CTAT]2 CAT [CTAT]8	1	0.005		35	[AAAG]16 [AG]12 [AAAG]13	1	0.02
D7S3048	16	[TATC]9 [TACC]7	1	0.005		36.2	[AAAG]19 [AG]13 [AAAG]11	1	0.02
	18	[TATC]12 [TACC]6	1	0.005		36.2	[AAAG]17 [AG]17 [AAAG]11	2	0.04
	20	[TATC]13 [TACC]7	2	0.01		37	[AAAG]16 [AG]16 [AAAG]13	1	0.02
	21	[TATC]11 [TACC]8 [CACC]2	1	0.005		37	[AAAG]18 [AG]12 [AAAG]13	1	0.02
	21	[TATC]12 [TACC]7 [CACC]2	4	0.02		37.2	[AAAG]17 [AG]15 [AAAG]13	1	0.02
	21	[TATC]10 [TACC]8 [CACC]3	1	0.005		39	[AAAG]19 [AG]13 [AAAG]14	1	0.02
	22	[TATC]14 [TACC]8	1	0.005		40	[AAAG]20 [AG]16 [AAAG]12	1	0.02
	23	[TATC]12 [TACC]6 [CACC]1 [TACC]2	1	0.005		40.2	[AAAG]20 [AG]13 [AAAG]14	2	0.04
		[CACC]2				40.2	[AAAG]18 [AG]21 [AAAG]12	1	0.02
	25	[TATC]14 [TACC]9 [CACC]2	1	0.005		41	[AAAG]21 [AG]14 [AAAG]13	1	0.02
	26	[TATC]15 [TACC]9 [CACC]2	1	0.005		42	[AAAG]22 [AG]16 [AAAG]12	1	0.02
D3S1744	16	[ATAG]2 ATG [ATAG]13 AG [ATAG]1	1	0.005	DYS587	23	[ATACA]17 [GTACA]1 [ATACA]1 [GTACA]1	2	0.04
DYS527a/b	18	[GAAA]11 [GGAA]7	1	0.01			[ATACA]1 [GTACA]1 [ATACA]1		
	22	[GAAA]14 [GGAA]8	1	0.01	DYS626	30	[AAAG]21 [AGAA]2 AGAG [GAAG]3	1	0.02
	25	[GAAA]18 [GGAA]7	1	0.01			[AAAG]3		
DYS458	21	[GAAG]1 [GAAA]20	1	0.02	DYS644	23.4	TTTTT [TTTTA]11 [TTTA]1 [TTTTA]11	1	0.02
DYS518	38	[AAAG]3 [GAAG]1 [AAAG]13 [GGAG]1	2	0.04	DYF387S1a/b	37.3	[AAAG]3 [GTAG]1 [GAAG]4 [AAAG]2	2	0.02
		[AAAG]4 N6 [AAAG]12 N27 [AAGG]4					[GAAG]1 [AAAG]2 [GAAG]11 [AAAG]3 AAG		
	41	[AAAG]3 [GAAG]1 [AAAG]12 [GGAG]1	1	0.02			[AAAG]10		
		[AAAG]4 N6 [AAAG]16 N27 [AAGG]4				38	[AAAG]3 [GTAG]1 [GAAG]3 AAAA [AAAG]2	5	0.05
	46	[AAAG]3 [GAAG]1 [AAAG]16 [GGAG]1	1	0.02			[GAAG]1 [AAAG]2 [GAAG]9 [AAAG]16		
		[AAAG]4				39	[AAAG]3 [GTAG]1 [GAAG]3 GAAA [AAAG]2	3	0.03
		[GAAG]1 AG [AAAG]17 N27 [AAGG]4					[GAAG]1 [AAAG]2 [GAAG]9 [AAAG]17		
	47	[AAAG]3 [GAAG]1 [AAAG]18 [GGAG]1	1	0.02	DXS10074	20	[AGAA]16 [AGAG]1 [AGAA]3	1	0.005
		[AAAG]4 [GAAG]1 AG [AAAG]16 N27			DXS10135	20	[AAGA]3 G [AAAG]1 GA [AAGA]15 AAGC	1	0.005
		[AAGG]4					[AAAG]1		
DYS645	6	[TGTTT]6	1	0.02		25	[AAGA]3 G [AAAG]1 GA [AAGA]19 AAGG	1	0.005
	11	[TGTTT]11	2	0.04			[AAGA]1 [AAAG]1	1	0.005

At the D18S51 locus, a 17.1 allele based on size has been previously noted; there is a lack of sequence information in the literature [43]. Despite the locus typically showing minimal sequence variation, the variant was also detected in this study, representing an insertion of “A” between [AGAA]5 and [AGAA]12. This proposition finds support in both the motif sequences and the sequences of the FRs, where an identical sequence is observed. For Y-STRs, a variant allele 37.3 on DYF387S1a/b was characterized by DNA sequence analysis and was found to contain a trinucleotide “AAG” insertion as follows: [AAAG]3 [GTAG]1 [GAAG]4 [AAAG]2 [GAAG]1[AAAG]2[GAAG]11[AAAG]3 AAG [AAAG]10.

Intriguingly, we found that the results for D9S925 locus in this study were discordant with those in other studies. Take the allele tatatatatgtctgt[CTAT]8 [CTAC]2 [CTAT]3 as an example, the nomenclature was 8 + 2 + 3 = 13. Although this motif sequence was consistent with the previously reported CE identification results [44], according to the ISFG recommendation [22] and STRiDER (https://strider.online/), the allele was 16 (recognized as [TATA]2 [TGTC]2 [TATC]8 [TACC]2 [TATC]2tat). Another discordance was observed at D14S608, resulting from different nomenclature methods between sequence-based alleles and the corresponding CE alleles, which was also reported in our previous study [12]. Lately, we recorded the sequence-based alleles of aforementioned two STRs following the ISFG recommendation. Consequently, great caution needs to be applied by forensic practitioners when integrating and comparing STR genotypes generated by different MPS platforms and CE.

The FR of 40 STRs, including 20 A-STRs (D10S2325, D11S2368, D13S317, D16S539, D17S974, D1S1627, D1S1656, D20S1082, D20S470, D20S482, D21S1270, D2S1338, D2S441, D3S3053, D3S3045, D5S818, D6S1017, D7S820, D9S2157, and vWA), 12 X-STRs (DXS10079, DXS101, DXS10146, DXS10148, DXS6800, DXS6803, DXS6809, DXS7132, DXS7424, DXS8378, DXS9902, and HPRTB), and eight Y-STRs (DYS388, DYS455, DYS531, DYS617, DYS626, DYS627, DYS713, and DYS720), contained a total of 55 SNPs and 13 InDels, demonstrating an increase in the number of unique alleles identified using MPS *versus* CE.

Indeed, we identified some rare variants in the FR of some loci (Supplementary Tables S2–S4). Additionally, Indels in the FR of some of the A-STR loci were so frequent that they could be observed in a significant proportion of sequence-based alleles. A repeat of dinucleotide “TG” insertion was observed in the downstream FR of D21S1270. Specifically, we found “TG”, “TGTG”, “TGTGTG”, and “TGTGTGTGTGTGTG” insertion in the downstream FR; two SNPs (rs61561022 and rs11910883) were also observed in this region; these insertions and SNPs result in the identification of 27 additional alleles based on the sequence polymorphism of D21S1270. In addition, D2S441 was shown to exhibit the higher proportion (85/200) of sequence-based alleles with flanking SNPs (GRCh38-Chr2: 68011829-C), and D6S1017 follows a similar pattern (GRCh38-chr6: 41709690-G, 40/200). Intriguingly, we found an SNP (GRCh38-chrX: 9271036-A) in the upstream FR of all alleles in DXS10148 locus, although this SNP was not recorded in the single nucleotide polymorphism database (dbSNP). The DXS6803 locus allele range consistent with previous reports [45, 46], and the divergence at this allele would suggest a “TGA” insertion between [TAGA]1 and [TAGA]12. Notably, we also found that two SNPs in the upstream FR almost appeared along with DXS6803, such as 10.3-GRCh38-chX:86431110-C, 86431140-T; 11.3-GRCh38-chX:86431110-C, 86431140-T; and 12.3-GRCh38-chX:86431110-C, 86431140-T.

### Forensic parameters

All autosomal loci and X-chromosome loci (females only), with either LB or SB allele data, were in HWE after Bonferroni correction (A-STRs: α′ = 0.05/66, X-STRs: α′ = 0.05/29). In terms of LD, no significant LD was detected in A-STRs or X-STRs for both LB data and SB data after Bonferroni correction (A-STRs: α′ = 0.05/2145, X-STRs: α′ = 0.05/406). The results of the HWE and LD assessment are shown in Supplementary Tables S5 and S6.

#### Autosomes

For A-STRs (Supplementary Table S5B), the most informative locus was D8S1132 (Heterozygosity (Ho) = 0.91), while the least information was obtained from D1S1627 (Ho = 0.48). When comparing Hobs values between LB and SB data, an average increase of 2.95% was observed. The four loci with the largest increases were D13S325 (15.00%), D11S4463 (14.00%), D2S1360 (13.00%), and D2S441 (11.00%). In descending order, the remaining 35 loci displayed increases ranging from 9% to 1%. This distribution pattern among loci is similar to that Guo et al. reported in the Asian population [10, 32]. With the exception of D1S1677 (GD = 0.6848; PIC = 0.6261), D20S1082 (GD = 0.6857; PIC = 0.6378), D2S1360 (GD = 0.6100; PIC = 0.6715), D5S2800 (GD = 0.6376; PIC = 0.6926), D1GATA113 (GD = 0.4983; PIC = 0.5508), D1S1627 (GD = 0.5428; PIC = 0.6004), D4S2364 (GD = 0.5719; PIC = 0.6473), TPOX (GD = 0.6039; PIC = 0.5366), and TH01 (GD = 0.6399; PIC = 0.5786), all the loci exhibited high levels of polymorphism (GD or Hexp >0.7 and PIC >0.6) for both SB and LB data. These findings are consistent with common observations in Chinese populations [31, 36, 47–49].

Forensic parameters for A-STRs were computed separately using allelic frequencies from LB and SB approaches (Supplementary Table S5A). Among the 66 STRs, the expanding diversity of SB alleles is evident in a noticeable increase in total discrimination power and a decrease in cumulative random match probability. Additionally, there was a substantial rise in combined parentage index (CPI) to 1.163 × 10^19^, ~146 times greater than the value obtained by length. Consequently, a clear upward trajectory was noted in combined power of discrimination (CPD), shifting from 1–9.456 × 10^−69^ to 1–2.411 × 10^−73^ (based on LB data and SB data, respectively), and in combined power of exclusion (CPE), ranging from 1–3.1955 × 10^−19^ (LB data) to 1–3.5814 × 10^−21^ (SB data).

#### X-chromosomes

For X-STRs, the frequencies of X-STR alleles in females and males were collectively analysed, as no significant differentiation was observed (*P* > 0.05). We calculated the forensic parameters using pooled allelic frequencies for the X-chromosome (Supplementary Table S6). Supplementary Table S6A demonstrates that, except for DXS6800, DXS7133, DXS7423, DXS8378, and GATA165B12 (with GD < 0.7 and PIC <0.6), all loci were highly polymorphic for both SB and LB data, which aligns with the findings of Guo et al. [10]. The average percentage of GD increase from LB to SB data was 1.60%, with the largest increases at DXS101 (13.90%) and the second at DXS6809 (6.00%). In total, the combined PD_F_, PD_M_, MEC_Desmarais_, and MEC_Desmarais Duo_ of the SB data were found to exceed 1–6.0890 × 10^−34^, 1–3.4167 × 10^−20^, 1–9.5178 × 10^−19^, and 1–1.06176 × 10^−13^, respectively (Supplementary Table S6), due to the increase in the number of unique alleles.

#### Y-chromosomes

For Y-STRs loci (Supplementary Table S7), the GD values ranged from 0.0404 (DYS472) to 0.9653 (DYS385a/b) for LB data and from 0.0404 (DYS472) to 0.9963 (DYF387S1a/b) for SB data. The percentage increase in GD values for SB data compared with LB data varied from 1.47% (DYS385a/b) to 30.93% (DYS437). Compared with LB alleles, SB alleles contributed to a 3.60% average GD increase, with >10% increases at DYS437 (30.93%), DYS455 (20.57%), DYS617 (19.68%), DYS388 (16.57%), DYS485 (15.75%), DYS531 (14.61%), DYS389II (14.20%), DYS713 (12.81%), DYS533 (12.57%), DYS520 (11.76%), DYS518 (10.61%), and DYS552 (10.20%). This finding is consistent with what Guo et al. [10] discovered in the northern Han population. Among these Y-STR loci, a total of 49 haplotypes were observed in both LB and SB data, resulting in an HD of 0.9996, and 48 of these haplotypes were unique (97.92%).

### Sequence variation at A-SNPs

Test for HWE was performed on the 132 A-SNPs; all SNP data satisfied HWE predictions following Bonferroni correction (α = 0.05/132). No significant LD was detected in 132 A-SNPs (8 646 pairs of comparison) after Bonferroni correction (α′ =0.000005783 0). The results of the HWE and LD assessment are shown in Supplementary Table S8. Supplementary Table S9 displays the allelic frequency and forensic parameters for the A-SNPs. The A-SNP with the range of discrimination power was rs740910 (DP = 0.228 6) to rs576261 (DP = 0.663 4). The A-SNP with the highest discrimination power was rs576261 (DP = 0.663 4) and the combined match probability was 8.431 8 × 10^−52^. Furthermore, the A-SNP with the highest PE was rs9956753 (PE = 0.290 9), and the CPE was exceptionally high at 99.999 999 988%.

## Conclusion

Here, we report the MPS-STRs and SNPs profile data for the Han Chinese population by FGID-DNBSEQ-G99RS Platform. DNBSEQ-G99RS platform is BGI’s dedicated sequencing platform tailored for small to medium throughput applications, featuring a highly adaptable detection system. By employing cPAS and advanced DNB, the process entails iteratively ligating, circularizing, and cleaving template DNA to create a circular template. Subsequently, this circular template undergoes RCA progress, resulting in the generation of a significant quantity of concatemers, specifically the DNBs. These technologies enable the simultaneous sequencing of diverse genetic markers, encompassing STRs, SNPs, and mitochondrial markers [21, 50]. In comparison with the ForenSeq™ panel (153 loci, including 28 A-STRs, 7 X-STRs, 24 Y-STRs, and 94 A-SNPs) and the HID Ion AmpliSeq™ Identity Panel (124 SNPs, including 90 A-SNPs and 34 Y-SNPs), it offers a broader coverage of chromosomal genetic markers and enables the acquisition of more genetic information for individual and parentage identification [13, 51, 52]. The observed diversity of alleles, allelic frequencies, and variants from RR and FR were analysed and compared. As expected, forensic parameters significantly increased in CPD and CPE for A-STRs. There was a modest increase in CPD and combination MEC on X-STRs, and little increase in DC on Y-STRs, which clearly proves the advantages of MPS compared with CE.

Moreover, there are still potential limitations or challenges that may arise in this study. To conform with the backward compatibility of the CE typing system, ISFG’s recommended nomenclature incorporates repeated numbering information according to allele length. Discrepancies in SB allele naming emerge when employing various coordinates of sequence guides and analysis tools, such as some researchers in past SB allele studies adhered to ISFG’s nomenclature, while others opted for custom naming conventions. Such divergent naming practices may result in inconsistent allele calls across laboratories, adding complexity to precise allele naming across populations. In this study, we adopt the nomenclature recommended by ISFG. Forensic practitioners are suggested to be very careful when integrating and comparing STR genotypes produced by different MPS platforms and CE. This also indicates that there is room for further improvement in the FGID forensic four-in-one DNA typing kit’s corresponding analytical software.

In conclusion, with further data aggregation and extensive validation, MPS technology has the potential to generate high-resolution and high-quality datasets for human identification and population genetics studies and can serve as a valuable tool in forensic genetics and may complement routine casework.

## Supplementary Material

Supplyment_table-Typesetting_version_owae050
